# Gastrointestinal symptoms in HIV-positive kidney transplant candidates and recipients from an HIV-positive donor

**DOI:** 10.1038/s41598-021-92016-2

**Published:** 2021-06-15

**Authors:** C. J. Martin, F. J. Veldman, D. Labadarios, Z. Ebrahim, E. Muller, S. M. Kassier

**Affiliations:** 1 Department of Human Nutrition, Faculty of Health Sciences, Room 4-14, HW, Snyman Building (South), University of Pretoria, Bophelo Road, Gezina, Private Bag X323, Pretoria, 0031 South Africa; 2 Department of Human Nutrition and Dietetics Registry, Sefako Makgatho Health Sciences University (Medunsa), P.O Box 60, Pretoria, 0204 South Africa; 3 Professor Emeritus, Division of Human Nutrition, Faculty of Medicine and Health Sciences, Stellenbosch University, Private Bag X1, Matieland, Western Cape 7602 South Africa; 4 Division of Human Nutrition, Faculty of Medicine and Health Sciences, Stellenbosch University, Private Bag X1, Matieland, Western Cape 7602 South Africa; 5 Department of Surgery, University of Cape Town, Private Bag X3, Rondebosch, Cape Town, Western Cape 7701 South Africa; 6 Department of Dietetics and Human Nutrition, School of Agricultural, Earth and Environmental Sciences, College of Agriculture, Engineering and Science, University of KwaZulu-Natal, Private Bag X01, Scottsville, Pietermaritzburg, 3209 South Africa

**Keywords:** Gastroenterology, Medical research, Signs and symptoms

## Abstract

Gastrointestinal symptoms (GIS) are common in kidney transplant candidates and recipients and may be worsened by HIV. Objective: To determine the frequency and severity of GIS in HIV-positive kidney transplant recipients from HIV-positive donors, and those waiting to receive one. A GIS rating scale (GSRS) was completed by 76 participants at baseline and at 6 months. GIS frequency was defined as having at least one symptom (GSRS > 1). Severity was indicated by the GSRS score. Transplant candidates: GIS frequency was 88.9% and 86.3% at baseline and 6 months respectively. Indigestion was the most frequent (79.6% and 66.7% at baseline and 6 months), and severe GIS (GSRS 2.3). Women reported global mean (*p* = 0.030) severity significantly more than men. Transplant recipients: GIS frequency was 95.2% and 76.2% at baseline and 6 months respectively. At both assessment points, indigestion occurred most frequently (85.7% and 61.9% respectively). Highest GSRS was reported for indigestion at baseline (2.33) and at 6 months (1.33). Waist circumference (WC) was positively associated with the severity of constipation GSRS. GIS are common in both groups, especially indigestions. WC in transplant recipients should be monitored.

## Introduction

Patients with impaired kidney function very often experience gastrointestinal symptoms (GIS) at all stages of chronic kidney disease (CKD)^1^. Symptoms begin early, appearing well before end-stage renal disease (ESRD), at stage 3 (eGFR 45 ml/min/1.73 m^2^), and become increasingly burdensome as kidney function declines^2^. Uraemia and dialysis predispose patients to gastrointestinal (GI) mucosal lesions and functional disorders^3^ that may or may not cause GIS^4^. In two recent studies, ESRD dialysed and non-dialysed patients reported a prevalence of GIS of 61.6% to 81.0%^5,6^. Following a transplant, renal function is restored, however, the occurrence of GIS remains frequent and is often an under-estimated problem^7^. At this point however, GIS is largely attributable to opportunistic infections and immunosuppressant therapy^8,9^.

CKD often coexists with other illnesses that affect the GIT through the disease process and its treatment. In HIV-positive individuals, replication of the virus in gut-associated lymphoid tissues^10^, pharmacological side-effects and opportunistic as well as non-opportunistic infections^11^, are known determinants of GIS. Resultantly, GIS may present at any time, in any area of the GIT^12^. Despite a paucity of data, in all probability, the prevalence of GIS among HIV-positive patients with ESRD may be higher than among uninfected patients with HIV.

Regardless of aetiology, the severity of GIS range from mild to severe, thereby compromising nutritional status^13^, psychological health^14^ and quality of life^7^. More importantly, GIS could be indicative of high risk complications such as upper gastrointestinal intestinal (UGI) bleeding in dialysed patients^15^ or graft failure in transplant recipients^16^.

Individually, both CKD and HIV have a significant impact on the GIT. However, the nature of GIS in ESRD together with HIV is unknown. For this reason, the primary aim of this study was to describe GIS in terms of frequency and severity as experienced by HIV- infected pre- and post-transplant recipients at baseline and 6 month follow-up. In addition, the study investigated the relationship between GIS and selected nutritional and clinical parameters.

## Methods

### Participants

Groote Schuur Hospital (GSH) in Cape Town, South Africa runs the HIV “positive-to-positive” kidney transplant programme. The programme extends nationally, with candidates and recipients resident across the country. Prospective transplant candidates who meet the inclusion criteria receive dialysis in their home province until a donor becomes available. Candidates then travel to GSH for the transplant, before returning home. For the purposes of this study, the most recent list of transplant recipients and potential candidates was obtained from GSH. The number of candidates and recipients in this programme were still small, but at the time represent 100% of the global population of this unique group. There were 92 prospective participants (68 candidates, 24 recipients).

**Figure 1:**
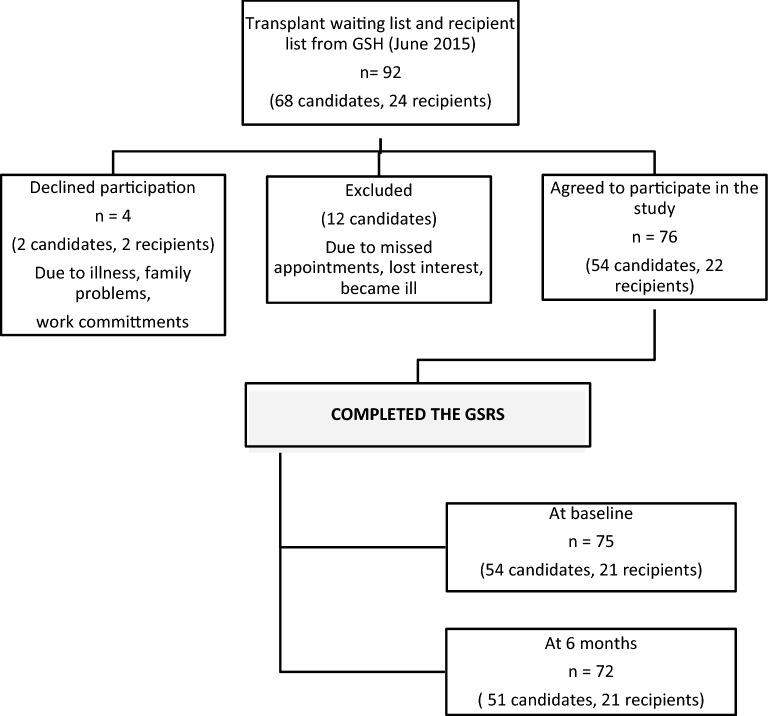
Patient participation flow chart.

Figure 1 indicates an overview of participant enrolment. Patients were contacted by phone or in outpatient clinics. Patients did not qualify for participation if they were severely ill, were not contactable, were uncooperative or missed several interview appointments (typically more than two without reason). Seventy-six patients agreed to participation. Written informed consent was obtained after the purpose of the study and practical implications were explained to them. They were assigned to two categories namely (i) HIV-positive transplant recipients who received a kidney from a HIV-positive donor (22 recipients); and (ii) HIV-positive transplant candidates who were on the waiting list to receive a kidney from a HIV-positive donor (54 candidates).

From June 2015, data was collected over one year with participants being followed up across six provinces. Assessments were conducted at two time points namely baseline and at 6 months follow-up.

### Anthropometry

Weight (WT), height (Ht) and waist circumference (WC) measurements were taken according to the National Health and Nutrition Examination Survey (NHANES) guidelines^17^ by a qualified dietitian. The mean of three readings were used for data analysis. Weight was determined post dialysis. BMI was classified according to the World Health Organization (WHO) categories as kg/m^2^: Underweight (< 18.5), Normal (18.5–24.9), Overweight (25.0–29.9), Obese Class I (30.0–34.9), and Obese Class II (35.0–39.9)^18^.

### Measurement of gastrointestinal symptoms

The Gastrointestinal Symptom Rating Scale (GSRS) was used to determine the frequency and severity of GIS^19^. Although originally designed for GIS assessment of gastrointestinal diseases, it has been used in all stages of CKD including dialysis^14^ and transplant recipients^20^. It consists of 15 items that are collapsed into 5 symptom subscales viz; abdominal pain (abdominal pain, hunger pain and nausea), reflux syndrome (heartburn and acid regurgitation), diarrhoea syndrome (diarrhoea, loose stools and urgent need for defecation), indigestion syndrome (borborygmus, abdominal distension, eructation and increased flatus) and constipation syndrome (constipation, hard stools and a feeling of incomplete evacuation)^19,21^.

GIS Frequency: The frequency of GIS was defined as having at least one symptom or a GSRS score > 1^14,21,22^.

GIS Severity: To determine the severity of a symptom, each question is rated using a seven-point Likert Scale ranging from one (no discomfort at all) to seven (severe discomfort) to obtain a total score ranging from 15 (minimum) to 105 (maximum) or mean values between one and seven. The combined severity scores of the five subscales, are presented as a global mean score and a mean score per subscale. Higher GSRS scores are indicative of a higher symptom burden. GSRS severity scores were correlated with patients’ clinical, demographic and nutritional parameters.

### Statistical analysis

Data was analysed using the Statistical Package for Social Sciences (SPSS®) version 25.0. Means and standard deviation were calculated for all continuous variables, and frequencies with percentages were determined for categorical variables. The means of groups were compared using the independent samples t-test. Cronbach’s alpha was used to determine the internal reliability of the GSRS. Spearman’s correlation was used to determine the relationship between GSRS subscales and clinical and nutritional variables. A *p* value of < 0.05 was taken as statistically significant.

### Ethical approval

Ethical approval for this study was obtained from the Biomedical Research Ethics Committee (BREC) of the University of Kwazulu-Natal. BREC is registered with the following: (i) South Africa’s Department of Health’s National Health Research Ethics Council (http://nhrec.health.gov.za) NHREC REC 290408-009). (ii) The US Office for Human Research Protections (http://www.hhs.gov/ohrp). (iii) Has Federal-Wide Assurance (FWA), Assurance number 678, Institution number IORG 0000923, IRB number 00001293.

## Results

### Patient characteristics

As all 76 patients completed the GSRS at least once, at either time points, no participants were excluded. At baseline, one patient did not complete the GSRS and four did not complete it at 6 months follow-up for reasons that included hospitalisation, missed appointments and the demise of two participants. Of the 76 participants surveyed, 22 HIV-positive kidney transplant recipients received a kidney from a HIV-positive donor, while 54 HIV-positive patients were on the waiting list to receive a kidney from a HIV-positive donor. The latter group were managed with haemodialysis (HD) (n = 51) or peritoneal dialysis (PD) (n = 3).

Socio-demographic, clinical, and nutritional status characteristics of the study population are given in Tables 1 and 2. The study sample, who were predominantly black (93.4%) and male (60.5%), had a mean age of 43.6 ± 8.1 years. There were significantly more patients with diabetes in the dialysis group compared to the transplant group (29.6% versus 4.5%, *p* = 0.017). At 6 months WC was significantly larger than that at baseline (*p* = 0.013).

**Table 1:** Socio-demographic and clinical characteristics of the study sample.

Patient characteristics	Whole group N = 76 n (%)	Transplant (n = 22) n (%)	Dialysis (n = 54) n (%)
Age (years): mean ± SD	43.6 ± 8.1 range: 28.0–63.0		
**Gender**			
Male	46 (60.5)		
Female	30 (39.5)		
**Ethnicity**			
Black	71 (93.4)		
Coloured^a^	4 (5.3)		
White	1 (1.3)		
**Type of treatment**			
Transplant		22 (28.9)	
Haemodialysis			51 (67.1)
Peritoneal dialysis			3 (3.9)
Length of time on current treatment (years)		2.7 ± 2.3 range: 0.0–6.8	3.9 ± 3.0 range: 0.3–11.5
Chronic illness			
Diabetes		1 (4.5)*	16 (29.6)*
Hypertension		19 (86.4)	51 (94.4)
Hypercholesteraemia		1 (4.5)	3 (5.6)
CD4 (cells/µL)^b^		447.25 ± 282.70	382.12 ± 178.02
Viral load (copies /ml)^c^			
LDL		18 (94.7)	39 (79.6)
≤ 10 000		1 (5.3)	7 (14.3)
> 10 000		0 (0.0)	3 (6.1)

Data expressed as percentages or means with standard deviation.

LDL: lower than detectable limit.

*Significant difference in the number of patients with diabetes between transplant recipients and transplant candidates patients (*p* = 0.017).

^a^Coloured is the term used in South Africa denoting mixed racial ancestry.

^b^Transplant patients: n = 20, dialysis recipients: n = 52.

^c^Transplant patients: n = 19, dialysis patients: n = 49.

**Table 2:** Nutritional characteristics of transplant candidates and recipients (N = 76).

Nutritional characteristics	Transplant (n = 22)				Dialysis (n = 54)			
	n	Baseline	n	6 months	n	baseline	n	6 months
Albumin (g/L)	20	43.1 ± 4.1	22	41.3 ± 4.1	48	35.9 ± 4.5	52	37.2 ± 4.8
BMI (kg/m^2^)	21	24.5 ± 4.6	22	25.6 ± 5.8	52	26.3 ± 4.8	49	25.7 ± 4.8
Underweight				1 (4.5)		1 (1.9)		1 (2.0)
Normal		15 (71.4)		13 (59.1)		19 (36.5)		19 (38.8)
Overweight		2 (9.5)		2 (9.1)		20 (38.5)		20 (40.8)
Obese class I		4 (19.0)		6 (27.3)		11 (21.2)		8 (16.3)
Obese class II						1 (1.9)		1 (2.0)
WC (cm)	18	89.6 ± 13.1^a^	18	95.8 ± 12.3^a^	36	92.2	46	90.7 ± 4.2

Data expressed as percentages or means with standard deviation or frequency with percentages.

^a^WC is significantly larger at 6 months than at baseline, paired samples t-test: t(14) = − 2.861, p 0.013.

### Gastrointestinal symptoms

**Figure 2:**
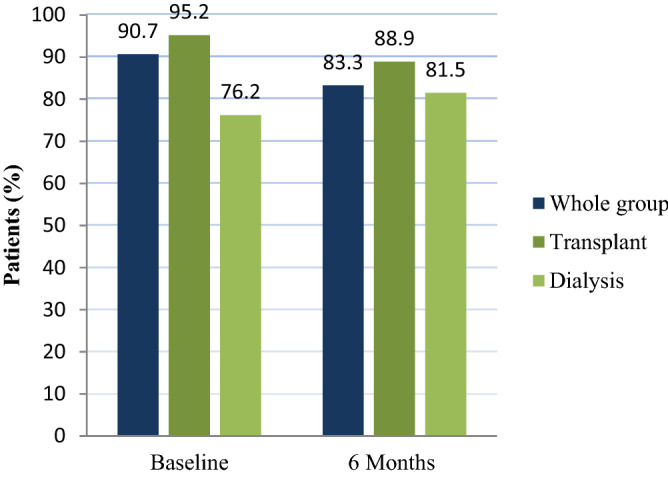
Frequency of gastrointestinal symptoms for the whole group and per treatment modality at baseline and 6-month follow-up (N = 76).

The frequency of at least one GIS (GSRS score of > 1) in the whole study sample is shown in Fig. 2, being 90.7% and 83.3% at baseline and 6 month follow-up respectively.

**Figure 3:**
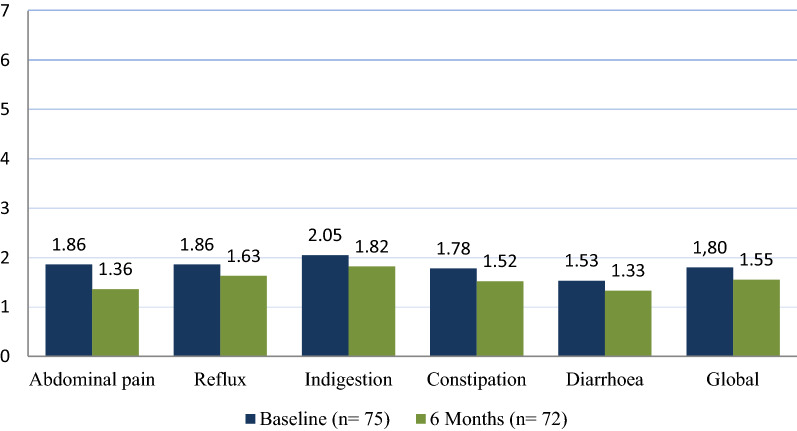
GSRS for the whole group across each subscale at baseline and 6-month follow-up.

The final Cronbach’s Alpha for the global mean at baseline and 6 month follow-up, was 0.813 and 0.862 respectively. GSRS for all GIS in the whole group (Fig. 3) was higher at baseline than at 6 month follow-up. At baseline, the global mean GSRS was 1.80 ± 0.76 and lower at 6 months at 1.55 ± 0.74. The individual GIS show a similar order of severity at each assessment time point. Indigestion and diarrhoea had the highest and lowest GSRS respectively.

### Frequency and severity of GIS in transplant candidates

**Figure 4:**
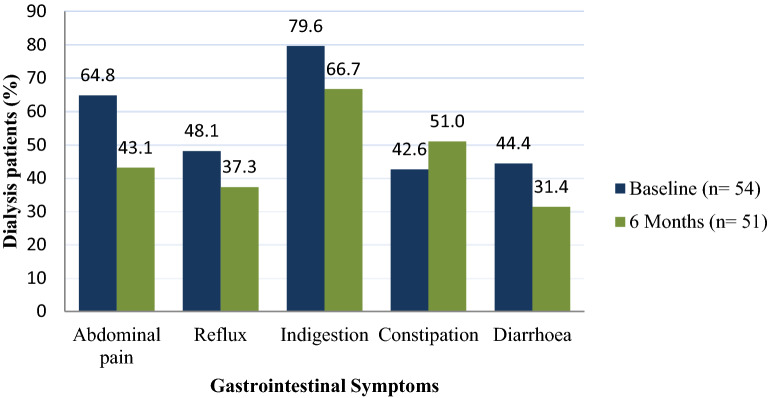
Frequency of gastrointestinal symptoms in transplant candidates at baseline and 6-month follow-up.

Overall, 88.9% of dialysed participants reported at least one GIS at baseline and 81.5% at 6 month follow-up (Fig. 4). At baseline, indigestion (79.6%), abdominal pain (64.8%) and reflux (48.1%) were the most commonly reported GIS, while diarrhoea and constipation were experienced to a lesser extent at 44.4% and 42.6%, respectively (Fig. 4). At 6 month follow-up, indigestion was still the most frequent GIS, albeit to a lesser extent (66.7%). However, more participants complained of constipation, increasing in frequency to 51.0%.

The GSRS scores indicated the severity of symptoms (Table 3) for each treatment group. The most severe GIS for PD patients (n = 3) was diarrhoea at 6 months (GSRS 4). For HD patients, indigestion was slightly more severe than the other GIS at both times (GSRS 1.67). Females had significantly higher median GSRS for several GSRS subscales as well as the global mean at baseline (*p* = 0.030).

**Table 3:** GSRS scores per treatment group at baseline and 6 month follow-up.

Subscale	Haemodialysis		Peritoneal DIALYSIS		Dialysis (HD + PD)		Transplant	
	Baseline	6 months	Baseline	6 months	Baseline	6 months	Baseline	6 months
	n = 51 (%)	n = 48	n = 3	n = 3	n = 54	n = 51	n = 21	n = 21
Abdominal pain	1.67 (4)^g^	1 (2.5)	2 (2.67)	1 (6)	1.67 (1)^a^	4 (6)^e^	1.67 (4.67)	1 (1)
Reflux	1 (5)	1 (4)	1 (4)	1 (5.5)	1 (1)	5 (5.5)	1 (4)	1 (5)
Indigestion	1.67 (6)^h^	1.67 (5)	2 (1)	1 (3)	1.67 (1.67)^b^	6 (5)^f^	2.33 (3)	1.33 (2.33)
Constipation	1 (6)	1.33 (6)	2 (4.5)	1 (1.67)	1 (1.33)	6 (6)	1 (2)	1 (2)
Diarrhoea	1 (3)	1 (4.5)	1 (6)	4 (5)	1 (1)^c^	6 (5)	1 (2)	1 (0)
Global mean	1.57 (3.14)	1.38 (3.62)	1.5 (3.5)	1.77 (3.62)	1.57 (1.38)^d^	3.93 (3.62)	1.86 (1.79)	1.15 (1.46)

GSRS scores.

^a^Significantly higher for females (mean rank = 32.66) than males (mean rank = 23.95), *p* = .040.

^b^Significantly higher for females (mean rank = 34.00) than males (mean rank = 23.03), *p* = .011.

^c^Significantly higher for females (mean rank = 32.27) than males (mean rank = 22.22), *p* = .022.

^d^Significantly higher for females (mean rank = 33.09) than males (mean rank = 23.66), *p* = .030.

^e^Significantly higher for females (mean rank = 30.85) than males (mean rank = 22.87), *p* = .025.

^f^Significantly higher for females (mean rank = 31.83) than males (mean rank = 22.24), *p* = .020.

^g^Significantly higher for females (mean rank = 30.83) than males (mean rank = 22.62), *p* = .045.

^h^Significantly higher for females (mean rank = 31.93) than males (mean rank = 21.85), *p* = .016.

In the transplant candidate group, Spearman’s correlations with GSRS (Table 4) were positive for the global mean score with the length of time on dialysis at baseline and 6 months (baseline rho = 0.287, *p* = 0.036 and rho = 0.440, *p* = 0.001). Age correlated negatively with GIS global mean (rho = − 0.338, *p* = 0.015).

**Table 4:** Correlations of GSRS scores with clinical and nutritional parameters in transplant candidates and recipients at baseline and at 6 month follow-up.

Variable	n	Global mean		Reflux		Indigestion		Constipation		Diarrhoea		Abdominal pain	
		Rho^a^	*p*	rho	*p*	rho	*p*	rho	*p*	rho	*p*	rho	*p*
**Dialysis**													
Duration of treatment													
Baseline	54	0.287*	0.036	0.279*	0.041								
6 months	51	0.44**	0.001			0.457**	0.001	0.3**	0.033				
Age													
Baseline	54												
6 months	51	− 0.338*	0.015	− 0.317*	0.023							− 0.354*	0.011
**Transplant**													
WC (cm)													
Baseline	18							0.471*	0.048				

*Correlation is significant at the 0.05 level (2-tailed).

**Correlation is significant at the 0.01 level (2-tailed).

^a^Spearman's correlation coefficient.

### Frequency and severity of GIS in transplant recipients

**Figure 5:**
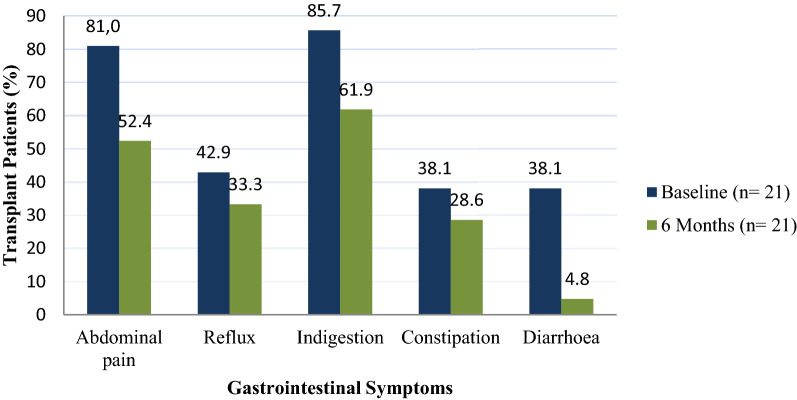
Frequency of gastrointestinal symptoms in transplant recipients at baseline and 6-month follow-up.

Over nine out of ten (95.2%) of the transplant group experienced GIS at baseline. However, the prevalence of symptoms decreased by 19.0% to 76.2%. The frequency of symptoms across the five subscales is depicted in Fig. 5. Transplant recipients reported indigestion as the most prevalent symptom at baseline (85.7%), this was followed by abdominal pain (81.0%), reflux (42.9%), with diarrhoea and constipation both occurring at a prevalence of 38.1%. At 6 month follow-up, frequency of GIS symptoms deceased by 19%, from 95.24 to 76.19%. The frequencies in each symptom category also decreased. Indigestion was the most frequently experienced GIS in the transplant group with 85.7% prevalence at baseline and 61.9% at 6 month follow-up. Only one participant reported diarrhoea (4.8%) at 6 month follow-up.

Indigestion was the most severe GIS with the highest median GSRS score of 2.33 at baseline and 1.33 at 6 months (Table 3). All GSRS were lower at 6 month follow-up with the global mean decreasing from 1.86 to 1.15. In the transplant group, WC was positively associated with constipation at baseline (rho = 0.471, *p* = 0.048).

## Discussion

To our knowledge, no data on GIS in a similar patient group exists. In the current study, the occurrence of at a least one GIS (GSRS > 1) in the total group, at baseline and at 6 months was high. At 90.7% and 83.3% respectively, this finding supports previous research that renal non -HIV patients experience a greater frequency of GIS than non-renal patients^23^ and the general population^14,24,25^.

### Transplant candidates

#### Frequency and severity of GIS

The frequency of GIS amongst dialysed participants was fairly consistent at both time points (88.9% and 81.5%). These values fell within the 76–90% GIS frequency range experienced by HD and PD populations elsewhere^26,27^. Across the five subscales indigestion, abdominal pain and, to a lesser extent, reflux occurred at higher freqency than constipation and diarrhoea at baseline. At 6 month follow-up however, more participants suffered from constipation, and with greater severity. These findings are in agreement with a systematic review of GIS in 30 studies conducted among 5161 HD and PD participants. Despite differences in methodology, these studies also reflected constipation, indigestion, abdominal pain and reflux as the most frequently reported GIS^3^. Constipation in particular, affects up to 71.7% of HD patients^28^ and is attributed to restrictive diets, medication, inactivity and ignoring the urge to defaecate whilst on dialysis^29^. Although constipation affected about half of the participants on dialysis, it was not the most bothersome GIS. Indigestion was the GIS of greater frequency and severity.

Indigestion, or dyspepsia is a common occurrance in the HD and PD population, with a frequency ranging between 30.0–72.3% and 31.5–93.1% respectively and is responsible for the regular consumption of acid suppressants in 41.0–76.4% of patients^30,31^. Endoscopy in dyspeptic patients shows upper gastrointestinal (UGI) pathology in 60.0–68.0% of patients, with erosive and ulcerative changes found in the stomach, oesophagus and duodenum. The causes of UGI morbidity are complex. In addition to risk factors in the general population^32^, CKD determinants include hypergastrinaemia, inflammation and high levels of ammonia^33^. Delayed gastric emptying or heparin use in dialysis^4,34,35^ adds to GIS such that dialysed patients may have a greater symptom burden than non-dialysed ESRD patients^14^. Within the dialysed group itself, PD participants (albeit only three), had more pronounced GIS than HD participants. This is a common^36,37^ but inconsistent finding^5^ related to the effects of the dialysate present in the abdomen^37^. Between the sexes, females reported significantly higher GSRS, similar to that observed in a Turkish HD group^38^. However, this is not exclusive to CKD. In the general population, women experience more dyspeptic and irritable bowel syndrome symptoms^39–41^ on account of gender specific psychosocial factors, hormonal activity, as well as anatomical and functional differences in pain transmission pathways affecting sensitivity^42,43^.

#### Correlations between GSRS severity scores, clinical and nutritional variables

This study examined the relationships between severity (GSRS scores), rather than frequency of GIS, with selected clinical and nutritional parameters. Although expected, no significant associations were found between GSRS and serum albumin. Lower serum albumin is likely due to underlying illness or inflammation, such as infections rather than nutritional status^44^, which could worsen the severity of GIS. Abdominal pain and reflux scores decreased with age, possibly due to the disinclination of older individuals to report symptoms. Furthermore, there appears to be an adaptation to the intensity of chronic symptoms as well as symptoms becoming less specific, and more vague with advancing age^45,46^.

GSRS scores were positively associated with the duration of dialysis. The increasing severity of indigestion, constipation and reflux with a longer period on dialysis, is not a universal finding^45^, as typically the opposite occurs. More GIS is noted at the start of PD^37^ and in HD, related to hypotensive episodes at HD initiation^47^.

### Transplant recipients

#### Frequency and severity of GIS

GI complications are a common occurrence following a solid organ transplant, potentially affecting any area of the GIT^9^. Severe complications are rare (10.0%), occurring primarily in the first year post transplant^48^. A transplant is expected to relieve GIS related to uraemia and dialysis, and explains the lower global GSRS scores in this study’s transplant candidates versus transplant recipients. However, for many transplant recipients GIS still persist, albeit with a lower level of severity. The transplant recipients in the current study had a high frequency of GIS at baseline (95.2%), similar to findings in European (88.3–92.0%)^7,22^ and African (96%) transplant recipients^49^. In a study by Ponticelli et al., with a cohort of 1130 kidney transplant recipients, patients demonstrated stable GIS throughout the year-long study period^7^. In contrast, the frequency of GIS dropped by 19.0% to 76.2% in the present study, for reasons that are unclear.

As was the case in the dialysis group, indigestion was a frequent symptom. It was the most severe at baseline and at 6 month follow-up, possibly due to underlying gastropathology. Dyspeptic transplant recipients have shown a high prevalence of erosive changes on endoscopy, mainly gastritis (78.6%), that could be present pre-transplant^50^ and/or is aggravated by immunosuppressants^8^. Tacrolimus, which has been linked to duodenitis^50^, forms part of the anti-rejection regimen in addition to mycophenolate mofetil (MMF), and prednisone^51^, and could be a contributing factor. It is also interesting to note that indigestion, together with abdominal pain and reflux, were the three most frequent GIS at baseline and 6 month follow-up, similar to a survey of 4232 transplant recipients across four north European countries^22^. Taken together, these three GIS are typical of gastro-oesophageal reflux disease (GORD)^52^, for which CKD, transplantation and anti-rejection medication are risk factors^53^.

Anti-rejection medication also increases the risk of infectious and non-infectious diarrhoea by increasing vulnerability to infectious agents and compromising gut mucosal integrity and function^9^. In 13 out of 25 (52.0%) transplant recipients with chronic diarrhoea, infections and drug-related colitis associated with MMF were identified via colonoscopy^47^, while diarrhoea was linked to the toxicity profile of Tacrolimus^54^. Despite the combination of these two drugs in the current study’s participants treatment regime, diarrhoea was not as bothersome as the other GIS. Diarrhoea affected eight transplant candidates (38.1%) and only one (4.8%) transplant recipient at baseline and 6 month follow-up respectively. Furthermore, the severity scores of diarrhoea were low (GSRS of 1.00). Earlier studies report the frequency of diarrhoea to be between 22.8 and 53.0% and GSRS scores of between 1.44 ± 0.88 and 1.80 ± 1.10 in transplant recipients^7,22^. In the majority of cases, diarrhoea is transient and resolved with appropriate pharmaceutical and dietary management^55^. This is probably the reason for the difference in frequency at baseline and then at 6 months.

#### Correlations between GSRS severity scores, clinical and nutritional variables

The significant increase in WC from baseline to 6 months amongst recipients most likely reflects a combination of greater dietary flexibility, immunosuppressants and lack of exercise. Significant associations between GSRS constipation scores with WC were identified at baseline in the transplant group. In the general population, obesity is a risk factor for GORD and erosive oesophagitis in the long term^25^, while central obesity is related to non-erosive oesophageal disease^56^. However, the association of obesity with constipation and functional dyspepsia is less clear^57^.

It would therefore be sensible to ensure weight maintenance and a WC at optimum values. In 332 non-CKD participants who participated in a weight intervention programme that targeted behaviour, diet, and physical activity, participants reported an 81.0% and 55.0% decrease and resolution of GIS respectively^58^. In other research, weight management was less likely to improve symptoms in 211 participants for which the BMI – reflux relationship was independent of diet and exercise^59^. This highlights the contribution of clinical, pharmaceutical and demographic factors to GIS.

### Gastrointestinal symptoms and HIV

The contribution of coexisting illness to GIS in ESRD was clearly applicable to this patient group. HIV has always been associated with GIS, and it was not uncommon for HIV-positive individuals to experience regular episodes of diarrhoea^60^. However, defining research by Mönkemüller et al. has shown a change in the pattern of GI manifestations since the HAART era. The occurrences of opportunistic infections have reduced^61^, while UGI manifestations have increased, and are associated with improved immunocompetence related to HAART^11^. HIV-positive Japanese patients, report higher UGIS severity scores than non-HIV infected patients^62^. Findings of mucosal changes such as gastritis (48%) and gastric erythema (45%)^63^, and reflux, *H pylori* infection, and GORD have increased^11^. In all probability, these would aggravate UGI pathology of ESRD, and could underlie the higher frequency and severity of indigestion compared to the other GIS in the current study sample.

This study has several strengths. It is the first to investigate the frequency and severity of GIS in pre- and post-kidney transplant recipients infected with HIV. Secondly, the GSRS which has been previously validated in South Africa and elsewhere^64^ encompasses a range of symptoms applicable to the upper and lower GIT. Thirdly, despite the small study sample, the findings of this research are still generalizable as the majority of patients on the transplant lists were included in this study, and as such, are a fair representation of this group. A study limitation in this regard is that the number of PD patients (n = 3) is extremely small. Thus, the power of the statistical analysis using this group is severely limited. Hence, correlations were done using PD and HD combined into a single group (n = 54). For future studies though, PD and HD patients should be considered separately as the former is likely to have a greater influence on GIS. The lack of information on medication used to relieve GIS, as well as detailed renal function parameters, which would have benefited the analysis of the study results, is also a drawback. The study design, which provides a snapshot of GIS at two assessment points is suitable for prevalence studies, but limits the exploration of causal relationships^65^. Furthermore, conducting the assessments before and after a transplant would have been preferable. However, some patients wait years for a kidney to become available. Unfortunately, for this study, resources and finances were only sufficient for a 6-month data collection period.

Finally, this study did not have a control group to compare GIS with and without the presence of HIV, but should be considered in future research, along with a longer follow-up period to provide better insight into whether the symptoms documented are pervasive or transient.

In conclusion, this research contributes to the body of evidence on GIS experienced by kidney transplant candidates and recipients but extends to an understanding of these symptoms among those infected with HIV. The data confirm a high prevalence, but low severity of GIS in both treatment groups, although similar to that documented for non-HIV infected dialysis and transplant recipients. Indigestion was a bothersome GIS in the whole group at both time points, while those on dialysis experienced a greater frequency of constipation at 6 month follow-up. A comparison of GSRS scores between groups showed higher severity scores in transplant candidates, and Spearman’s correlations with specific GIS were positive for duration of dialysis and negative for age. In the transplant group, specific GIS were positively associated with WC.

Both kidney transplants and dialysis are major medical interventions that are often accompanied by complications, and frequent hospitalisation. However, GIS (especially if they are chronic and low grade), may be discounted by patients and clinicians until they become severe and debilitating. Major gastrointestinal complications are rare, but do occur. The GSRS is a quick, simple, and cost effective monitoring tool that can be used for early identification, or progression, of GI manifestations.
